# Real-Time Monitoring System for Shelf Life Estimation of Fruit and Vegetables

**DOI:** 10.3390/s20071860

**Published:** 2020-03-27

**Authors:** Roque Torres-Sánchez, María Teresa Martínez-Zafra, Noelia Castillejo, Antonio Guillamón-Frutos, Francisco Artés-Hernández

**Affiliations:** 1 Systems and Electronics Division Group, ETSII, Universidad Politécnica de Cartagena, 30202 Cartagena, Spain; mtmz87@gmail.com; 2 Postharvest and Refrigeration Group, ETSIA, Institute of Vegetal Biotechnology, Universidad Politécnica de Cartagena, 30202 Cartagena, Spain; noelia.castillejo@upct.es (N.C.); fr.artes-hdez@upct.es (F.A.-H.); 3 Models and Systems for Signal Processing, Time Series, Astronomy and System Reliability Group, ETSII, Universidad Politécnica de Cartagena, 30202 Cartagena, Spain; antonio.guillamon@upct.es

**Keywords:** cold-chain, wireless sensor networks, traceability, quality predictive modelling, postharvest, prevention of food losses

## Abstract

The control of the main environmental factors that influence the quality of perishable products is one of the main challenges of the food industry. Temperature is the main factor affecting quality, but other factors like relative humidity and gas concentrations (mainly C_2_H_4_, O_2_ and CO_2_) also play an important role in maintaining the postharvest quality of horticultural products. For this reason, monitoring such environmental factors is a key procedure to assure quality throughout shelf life and evaluate losses. Therefore, in order to estimate the quality losses that a perishable product can suffer during storage and transportation, a real-time monitoring system has been developed. This system can be used in all post-harvest steps thanks to its Wi-Fi wireless communication architecture. Several laboratory trials were conducted, using lettuce as a model, to determine quality-rating scales during shelf life under different storage temperature conditions. As a result, a multiple non-linear regression (MNLR) model is proposed relating the temperature and the maximum shelf life. This proposed model would allow to predict the days the commodities will reduce their theoretical shelf-life when an improper temperature during storage or in-transit occurs. The system, developed as a sensor-based tool, has been tested during several land transportation trips around Europe.

## 1. Introduction

Nowadays there is a synergistic effect between the importance of maintaining the quality of perishable products and market progress. A critical measure that should be immediately taken is to reduce the huge amount of postharvest losses, which are reported to be 1.3 billion tons a year, which represents 33% of the production according to FAO [1]. There is still a lot of work to be done, by all agents in the different sectors involved in the supply chain [2], in order to reduce such losses and better preserve the quality and safety of horticultural commodities after harvest. Shelf life is usually defined as the time during which a food product remains safe according to microbiological standards and retaining a desired sensory, physico-chemical and nutritional quality [3]. Food quality is commonly modelled using the Arrhenius equation. However, generic approaches are time-consuming when many experiments are needed. Therefore, a real-time measuring system supported by prediction models is required [4]. Nowadays, the support of information and communication technology (ICT) has opened a whole range of possibilities to design shelf life prediction models with measuring devices [5]. 

Storage or in-transit temperature is the most important environmental factor affecting perishable food quality [6]. Therefore, the actors involved in the supply chain must deliver the commodities preserving the cold chain to avoid inappropriate food loses and waste, while preserving quality and safety [7]. By controlling this parameter, a longer shelf life for perishable food products will be ensured, but it represents a difficult puzzle to solve due to the many factors involved [7]. In this work, lettuce has been selected as a model for the study of a quality loss estimation model depending on the storage temperature. Several authors [8,9,10,11] define the optimal storage temperature of lettuce within the range of 0–2 °C. However, the land transport stage of this product is usually conducted between 4 and 5 °C and the air conditions are not isothermal during the cold chain. Therefore, the shelf life of the transported commodities is usually reduced. A previous work related to fresh-cut lettuces has proposed shelf life models performing tests to determine the quality evolution of the fresh-cut lettuces with temperature changes [12].

Temperature monitoring systems are required by the receptors of the transported commodities to supervise perishable food quality in the supply chain [13]. Such systems will involve time-temperature integrators throughout the cold chain than can be used to estimate the shelf life and the expiration date must be associated with their thermal history [14]. Time Temperature Indicators (TTI) or small card-shaped dataloggers (which information is downloaded at destination) are normally used as temperature measuring systems [15,16]. Today’s technology provides advanced measurement platforms that allow us to measure the temperature at several locations with wireless data-reading devices. Such platforms must be flexible to adapt themselves to the different logistic conditions of perishable goods transportation and, in some devices, the data can be accessible by the agents at any time using cloud data systems.

Several platforms for wireless temperature measuring of commodities can currently be used. The solutions available make it possible to measure at different points in order to determine the variations that may exist in the thermal conditions of the commodities. In an example, during transport, air and product temperatures do not have the same dynamics, so fluctuations will differently affect both [17,18,19].

Radio Frequency Identification (RFID) solutions allow a wide deployment of sensors throughout the load and can be used up to battery-less operation [20]. A systematic review covering the 2004–2018 period was conducted in [21], citing the advantages and disadvantages of this technology and its practical implementation in temperature monitoring. 

Wireless sensor networks (WSN) deployments are also widely employed since they allow multi-hop node architectures, which solves coverage problems among nodes at times. Zigbee [22] or Bluetooth low energy (BLE) [23] have been used by some authors to monitor the conditions of products. Recently LoRa^®^ devices have been introduced into smart refrigeration solutions to track food temperature in the supply cold chain [24]. However, to optimize the destination or use of perishable foods in the event of changes in the thermal conditions of transport, it is necessary to implement a real-time tracking and monitoring system [7]. Some RFID, i-Button or Zigbee-based sensors require special equipment to read the data measured, which makes it more complicated to implement a real-time tracking and monitoring system [19].

Although the thermal conditions of the products being transported can vary depending on many issues such as the type of product, its packaging or the load density, among others, installing a measuring device on each box or pallet to measure the temperature is not realistic nor practical [19]. The current thermal air control during transportation, in the worst case, will allow reuse of the products, due to the rejection rate in warehouses (5%). Currently, many products are rejected due to the thermal records of the storage/transport air conditions. Land transportation systems do not measure the pulp temperature of fruits. Only some refrigerated containers can have up to three pulp temperature measurement probes, which are basically used to carry out cold quarantine treatments when exporting to countries that require it, but not as a usual procedure for measuring the temperature of the product during transportation. Moreover, due to transportation systems are not designed to efficiently cool down the commodity´s temperature, such products should be loaded in the container/truck at the set-point temperature. The refrigeration unit of the transportation system will then compensate the heat coming from the exterior by transmission or irradiation, and the respiration heat produced by the load [25].

Due to air thermal dynamic is higher than that of the commodities, since they have a ‘thermal inertia’, air temperature changes much faster than that of the product [26]. For this reason, the use of air temperature measurement systems requires appropriate statistical indicators that will allow the integration of differences in the dynamics between air and product temperatures [18].

Modelling methods such as accelerated shelf life testing (ASLT) [27], or the multivariate accelerated shelf life testing (MASLT) [28] based on principal component analysis (PCA) [27], have been proposed for shelf-life modelling. Recently, artificial neuronal networks (ANNs) have been regarded as a flexible modelling tool, because neural networks can model any type of relationship within the data with high accuracy [29]. As a recent evolution, machine learning (ML) techniques are used with artificial neural networks in the field of agricultural processes both in irrigation management [30] and in postharvest, specifically for shelf life prediction [18,31]. In [22], a specific ML is designed for the management of the cold chain for perishable food products. The system is implemented based on the WSN and TTI features. The intelligent container uses sensors and cognitive systems based on ML to estimate quality loss [32]. However, the prediction of quality loss of perishable products is a very complex process where the perception of final quality depends on a subjective assessment. For that reason, the result obtained by a system able to predict the remaining shelf life needs a human verification process. This verification process has to be carried out at the limit of the commercialization of the commodities, which makes it difficult to obtain tests to design and validate a representative model.

The main objective of this work is to propose a methodology able to predict shelf life losses of perishable commodities during the different cold supply chain stages (from harvest to the retail sale destination), based on sensory and physico-chemical quality attributes. To achieve this purpose, a portable, flexible and stand-alone monitoring system able to measure at multiple points with real-time communication through a standard Wi-Fi wireless technology has been developed [33,34]. A multiple non-linear regression (MNLR) model that correlates the theoretical shelf life (SL) and the temperature during cold storage and/or transportation, has been proposed. This model gives an approximation of the shelf life reduction during the whole supply chain. 

## 2. Materials and Methods

Lettuce was the commodity selected for the study as a model. This is due to the great trade importance of this vegetable and also to the physiological disorders that occur when stored under improper postharvest conditions of temperature, relative humidity, ethylene and CO_2_ [35]. Iceberg lettuce (*Lactuca sativa’*) was used for the trials. The Region of Murcia produces 51% of the total iceberg lettuce produced in Spain, 85% of which is exported to European countries [36].

**Figure 1:**
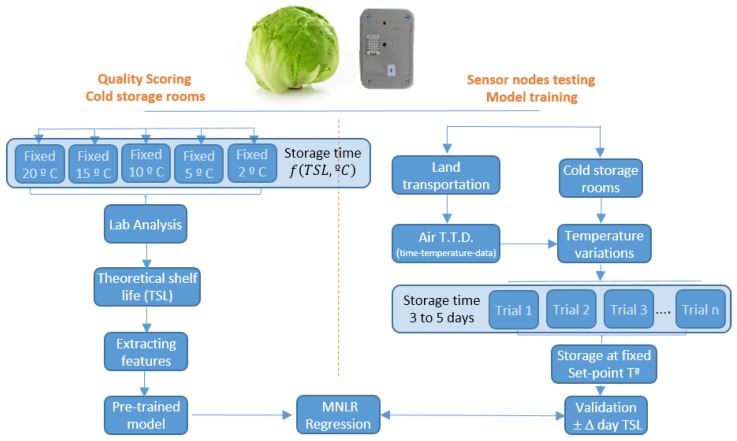
Flowchart of the performed tests.

Two experimental trials have been performed to determine the validity of the presented system. Firstly, a quality test was conducted in the cold storage rooms and laboratories of the university to determine the shelf life of the lettuces at different storage temperatures. In a second test, the sensors of the presented system were installed in refrigerated trucks, fully loaded with iceberg lettuce during commercial trips, and the temperature variations were evaluated. Afterwards, such real conditions were replicated in our cold storage rooms, where the lettuces shelf life was therefore tested and validated. Figure 1 shows the flowchart of the described process. To determine quality losses according to the storage temperature, the lettuces were stored in five cold storage rooms under different temperatures: 2, 5, 10, 15 and 20 °C. Quality degradation was evaluated by a trained panel in sensory analysis discrimination using a score according to its overall quality [37,38,39] The tests performed, allowed to obtain a correlation between the storage temperature and the shelf life of the product, defined as the minimum quality to be sold. However, such quality tests have been performed at constant temperatures and it is necessary to evaluate and predict the quality during the different steps of the cold chain, where the temperature is not constant. Therefore, the evaluation of the temperature variation must be related to the quality of the commodity by an indicator (feature) able to correlate the temperature during all postharvest steps until retail life [40]. 

In the second experimental trial, the sensors were installed in several refrigerated trucks containing lettuce during commercial trips, to check and debug the operation of the sensor-based system and the robustness of communications. In Section 2.3, the installation and methodology of the process are detailed. 

Mathematical models would be able to predict quality losses using the registered temperature throughout storage and/or transportation. As the data received during the transportation are time-temperature series, such losses will be related as the estimated time for the product to maintain a minimum quality (score) to be sold. In addition, it determines how long the vegetables can remain under such temperature conditions. This estimation could be scalable to the whole supply chain, from harvest to the retail sale store. However, this categorization would not be efficient enough due to the amount of information registered and involved. The data needed to build the estimation model needs to be validated by ‘an expert’, which means performing multiple product quality verification tests. In this work, land transportation simulations have been performed in cold storage rooms due to the great economic and time costs of the validations at the destination.

The materials and methods described in this paper have been divided into three sections: the equipment designed and used for the continuous monitoring of variables during transport, the physico-chemical and sensory quality tests performed on the commodity to determine its shelf life according to storage temperature, and the mathematical methods used to represent the estimation model.

### 2.1. Monitoring System

The patented monitoring system is based on the real-time control of the most influencing environmental variables to estimate the shelf life of the products during the whole supply chain. 

**Figure 2:**
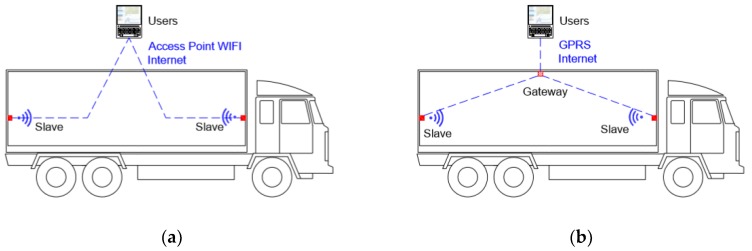
(**a**) Slave sensor nodes can use any WI-FI infrastructure to send the recorded information. (**b**) Gateway sensor node performs measurements like the slave but also generates a portable WI-FI infrastructure, sending the information of both the slave nodes and its own to the web server through the internet using commercial communication network (GPRS, SigFox or LTE).

Small, flexible and a long-life battery are the main characteristics of the system nodes, which can be divided into two types depending on the role in the system: ‘Gateway’ and ‘Slave’. All of them can monitor, process, save, and wirelessly transmit the data to a web server where the users can consult them in real-time and with programmable sampling time. Besides, the gateway transmits the geolocation. The different roles between the gateway and slave sensor nodes lie in the communication connection, as shown in Figure 2. 

The system has been developed to facilitate the measurement and access of the conditions of perishable products at any stage and location of the product in the post-harvest chain. For this reason, a solution based on a Wi-Fi 802.11 communication standard has been adopted and is present almost everywhere. In this way, the sensor nodes can send the registered information in real-time using these networks along with the different steps of the supply chain (collection, packing, transport, logistic reception, distribution and grocery store). The main motivations for the selection of this type of communication are based on:

(1) Flexibility: Wi-Fi networks based on the 802.11 standard protocol are present almost everywhere. Nowadays, it is common to find in cars, trains, buses, etc., own systems to generate WLAN access points using Wi-Fi. The smartphone we use every day allows us to generate shared connections to the internet using Wi-Fi. Even though it is not present in the companies, the acquisition and installation of routers with Wi-Fi access points are simple and very economical.
(2) Functionality: Slave sensor nodes can connect to the different Wi-Fi network during the cold chain using them to send the registered data. If there is no Wi-Fi coverage at any point in the post-harvest chain, the node stores the registered information to send it as soon as a network of the programmed list is available.
(3) Usability: We have developed the gateway sensor node to solve the lack of Wi-Fi coverage during, for example, land transportation. In addition, this node can perform measurements such as the slave sensor node. The communication with the server can be performed through GPRS, SigFox or LTE, according to the conditions or preferences of the users.

The hardware design of the sensor nodes must guarantee two basic requirements for appropriate working:

- They must be as economical as possible since, many times, some nodes will not be recovered. Therefore, we have selected the hardware platforms based on ESP32’s (ESPRESSIF SYSTEMS, Co., Ltd., Shanghai, China), that provides embedded Wi-Fi and BLE interfaces besides the several input/output pins for reading and control the sensors.
- They must consume very low power energy. Power autonomy enables them to be connected throughout the long journey. For that, a “Time Synchronizing” algorithm has been developed to reduce the slot time that the slave nodes are connected to the Wi-Fi network.

The environmental variables able to be measured and registered by the slave sensor node are: Temperature (−30 to 80 °C, LMT86 Texas Instruments, Dallas, TX, USA); relative humidity (0 to 100%, HIH 5030 Honeywell, Charlotte, NC, USA) and luminosity (0 to 300 lux, NSL06S53 Advanced Photonics, Ann Arbor, MI, USA). Besides, the gateway sensor node can measure the following variables: CO_2_ (0 to 20,000 ppm, COZIR-GC16 CO_2_ Meter, Ormond Beach, FL, USA) and ethylene (0 to 400 ppm, M-10 Membrapor, Wallisellen, Switzerland).

Even though the nodes can measure many atmospheric variables, this paper is just focused on temperature as it is the most significant factor by far in the quality degradation kinetics of perishable products, intending to propose a prediction methodology which will apply to the other parameters in future researches. The size and flexibility of the nodes allow them to be placed anywhere, i.e., on the walls of the truck, on a pallet or inside the cardboards of commodities.

### 2.2. Laboratory Test

Three sets of tests have been carried out in the pilot plant of the Institute of Vegetal Biotechnology (IBV) of the UPCT using five cold storage rooms and the corresponding quality laboratory to determine lettuces quality according to the storage temperature. Slave sensor nodes were used for temperature registration. They were connected to the Wi-Fi network available. Respiration rates, physico-chemical and ‘human’ sensory analysis were conducted for several sampling days throughout a shelf life period. The data obtained have been used for scoring the overall acceptance.

#### 2.2.1. Plant Material.

Iceberg lettuce (*Lactuca sativa*) were provided by Fruca Marketing S.L. (Murcia, Spain) and the trials were conducted during February, March, April and November of 2019. The lettuce was field packed in 2 mm diameter perforated 33 × 23 cm bags and vacuum cooled once at the handling facility, as they are usually prepared for commercial export purposes.

In order to perform the laboratory tests, the lettuces were directly transported from the pre-cooling room at Fruca Marketing S.L. to the cold storage rooms at the IBV of the UPCT, where they were stored at five fixed temperatures (2, 5, 10, 15 and 20 °C). An initial analysis (day 0) was performed to check the quality at harvest.

For each one of the three tests set, five lettuces previously randomly numbered were extracted each sampling day for quality determination at each storage temperature as it is presented in Table 1. Such quality analyses were destructive, so lettuces were not back again to the storage room. Therefore, it did not affect the quality of the lettuce for further quality analysis in the following days [41]. The recommended shelf life for iceberg lettuce is presented in Table 1 which is based on our preliminary unpublished trials and are in accordance with shelf life recommendations by UCDavis (CA, USA) [35]. 

#### 2.2.2. Respiration Rates

The respiration rate was determined using a gas chromatograph (7820A, Agilent Technologies, Santa Clara CA, USA), equipped with a thermal conductivity detector (200 °C), oven (80 °C), injector (120 °C) and with a Hayesep Q column (Teknokroma, Barcelona, Spain). Air and H_2_ were used as gas carriers at 30 and 3 mL min^−1^. For each temperature (2 °C, 5 °C and 10 °C), three replicates were analysed. One head of Iceberg lettuce (515 ± 60 g and 470 ± 53 g, respectively) was placed into 3.9 and 10.8 L hermetic plastic buckets, respectively. The results were expressed as mL CO_2_ kg^−1^ h^−1^.

#### 2.2.3. Physicochemical Analysis

Weight loss was determined in % as:

% = [(Initial weight − Final weight) × 100]/Initial weight(1)

Total Soluble Solids Content (SSC) was determined by a digital hand-held refractometer (Atago N1; Tokyo, Kanto, Japan) at 20 °C and expressed as °Brix (g sugar equivalents 100 g^−1^). A pH-meter (Basic20, Crison, Barcelona, Spain) was used to determine the pH. Titratable Acidity (TA) was determined by titration of 2 mL of lettuce juice plus 48 mL of distilled water with 0.1 M NaOH to pH 8.1–8.2 (T50, Metter Toledo, Milan, Italy) and expressed as g citric acid L^−1^). Colour was determined using a colourimeter (Chroma Meter CR–400, Minolta, Tokyo, Japan) calibrated with a white reference plate (light source C), 8 mm viewing aperture. Three colour readings were conducted in two different areas of the external leaf and the cut area and all measurements were averaged. Measurements were recorded using the standard tristimulus parameters (L*, a*, b*) of the CIE Lab system. Total colour differences (ΔE) throughout storage, compared to their respective initial values, were calculated according to (2). According to [42], firmness was recorded using a texture analyser (Ibertest eLib-5-W, Madrid, Spain) as the compression force (N) required to achieve a 5% deformation of the lettuce diameter at 100 mm min^−1^ speed. The results were expressed in N mm^−1^:

ΔE = [ΔL*2 + Δa*2 + Δb*2]^1/2^ (2)

#### 2.2.4. ‘Human’ Sensory Analysis

A ‘human’ sensory analysis was performed according to international standards [43] with different characteristics analysed concerning human perception and the bibliography [35]. The panel consisted of ten experimented trained assessors (aged 22–55 years) screened for sensory ability. A five-point scale of damage incidence and severity was scored for off-odours, freeze injury, mechanical damage and several disorders like russet spotting or pink rib (5: none; 4: slight; 3: moderate – limit of retail (LR) -; 2: severe; 1: extreme). Visual appearance, colour, compactness, flavour and overall quality were assessed using a 5-point hedonic scale of acceptability:

- Score: 5: excellent
- Score 4: good
- Score 3: fair – limit of retail (LR)
- Score 2: poor
- Score 1: extremely bad.

Testing was carried out in a quality analyses laboratory under artificial daylight-type illumination at constant temperature (20 °C) and air circulation.

#### 2.2.5. Statistical Analysis

The experiment was a two–factor (temperature × storage time) design subjected to analysis of variance (ANOVA) using Statgraphics Plus software (vs. 5.1, Statpoint Technologies Inc., Warrenton, VA, USA). Models were generated using R GNU statistical software. Statistical significance was assessed at the level *p* = 0.05, and Tukey’s multiple range test was used to separate means.

### 2.3. Land Transportation Trials

**Figure 3:**
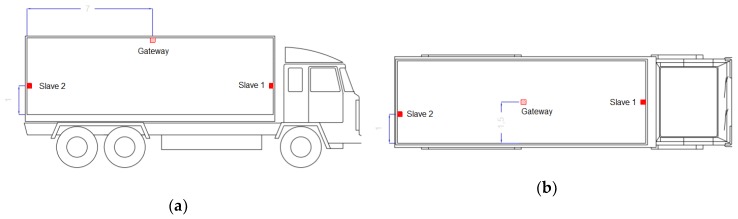
Distribution of the nodes during transportation (**a**) the longitudinal view and (**b**) the top view.

Fifteen trials were carried out on land transportation for three years due to the seasonality of the lettuce, in order to measure the temperature variations during real land transportation trips and to debug and test the proper working of the system developed. The monitoring system composed by one sensor gateway and two slave sensor nodes was installed on several commercial transport refrigeration trucks (Transportes Directos del Segura S.L., Lorquí, Spain and Transportes Mesa, S.L., La Palma, Spain) that departed from Murcia (Spain) to different European countries, such as England, Germany, Belgium and Netherlands. The distribution of the lettuces in the truck was as follows: thirty-three euro pallets with fifty-two cardboard boxes, size 60 × 40 × 15 cm, four boxes per layer and 13 stacks per pallet. Each box contained ten lettuces. The nodes were placed along with the truck, on the door, the roof and under the evaporator in order to observe the difference of air temperatures inside the truck (Figure 3). They were placed taking up minimum space of the total container volume and without interfering with the normal functioning. The air temperature was registered within the truck to analyse how the time-temperature variation of the air affects the lettuce. According to the lettuce exporter sector of the southeast of Spain, 5 ± 2% of exporting shipments are annually rejected due to poor quality and or inadequate storage or transportation conditions [1]. 

Six trials were performed in the facility of the cold room of the UPCT simulating land transportation conditions. In these trials, the temperature was randomly changed around set-point defined in the first 4–5 days, according to the air temperature variations observed in the real shipments. After these days, the lettuces were stored at constant set-point temperature until their quality analysis. Both in cold storage rooms and real land transportation experimental trials, the temperature was measured by the same sensor nodes.

### 2.4. Prediction Model

The prediction model tries to determine the reduction shelf life (SL) of the commodities when the temperature condition varies regarding the theoretical set-point. A supervised training process has been used. The quality reports carried out by a postharvest expert panel has been used as a ground truth. These quality tests were performed in several cold storage rooms of the IBV-UPCT, focused on classifying the items as a function of the five levels of acceptability (score) described in Section 2.2. These tests provided a correlation between temperature and time that establishes how long the commodities remain under the limit of marketability at a constant temperature condition.

However, this model aims to predict the quality loss in terms of retail shelf life reduction when the temperatures present variations regarding the set-point value. This is a common situation for perishable products from harvest to supermarket.

Different critical factors were extracted from the input data to perform adequately the prediction. These features (predictor variables X) have been tested in order to find good prediction model to determine the shelf life duration, in days (response variable Y) from the evolution of the temperature of the commodities in real-time during transportation:

- SP—Set-point temperature (Ideal temperature for commodities transportation).
- A—Area above the set-point measured by the sensor devices.
- ΔT—Maximum variation of the temperature.
- AT—Average temperature.

Two regression models have been tested, multiple linear regression (MLR) and multiple nonlinear regression (MNLR), with n predictor variables (1). The lettuces stored into the cold storage rooms were analyzed days before and after the shelf life recommendations according to similar temperatures [36] (Table 1), obtaining the response variable Y (shelf life duration) as the last day in which the quality of the commodities was categorized as score 3. 

The model are developed from a training set D = {X, Y} of S samples, which is composed of the predictor matrix, or also called design matrix, *X = [x_1_, . . . ,x_i_, . . . , x_S_]^T^* and the response matrix *Y = [y_1_, . . . , y_i_, . . . , y_S_]^T^. x_i_* is a column vector of K elements, that can contain all features at a given trial *i*.

For the MLR model:

*x_i_* = [SP;A;ΔT;AT]*^T^* (3)

and *y_i_* is a column vector of M elements, containing the corresponding days to be estimated at that trial *i*. Since in our application this is only the days that the commodity keeps the score above 3, *y_i_* is reduced to a scalar and M = 1:

y_i_ = *days with score 3*

We consider MLR as:

$$y_{i}=\beta_{0}+\beta_{1}x_{1i}+\beta_{2}x_{2i}+\beta_{3}x_{3i}+\beta_{4}x_{4i}+\epsilon_{i}\;i=1,\ldots ,n$$

In the MNLR model analyzed, we consider that the design matrix X is given by:

*x_i_* = [SP;A;ΔT;AT, SP^2^,A^2^, SP*A]*^T^* (4)

to determine the influence of quadratic terms, as well as the possible interaction between the set-point and the area above the set-point.

In this case, the MNLR model is:

$$y_{i}=\beta_{0}+\beta_{1}x_{1i}+\beta_{2}x_{2i}+\beta_{11}x_{1i}^{2}+\beta_{22}x_{2i}^{2}+\beta_{12}x_{1i}x_{2i}+\cdots +\epsilon_{i}\;i=1,\ldots ,n$$

Both models can be expressed in the matrix form as:

y=Xβ+ϵ

applying the least-squares criterion, we must estimate those *β* values that minimize the mean square error, i.e.,:

$$\overset{n}{\underset{i=1}{\sum }}\epsilon_{i}^{2}=\epsilon^{\prime }\epsilon ={\left(y-X\beta \right)}^{\prime }\left(y-X\beta \right)$$

This criterion determines those models that maintain a high explanatory level and contain regressors with statistically significant active influence on the response variable without collinearity issues.

## 3. Results

### 3.1. Laboratory Test

Significant differences in respiration rates at different temperatures were found. The higher the temperature the higher was the respiration rate of the lettuce as it was predicted (Table 2).

The physicochemical parameters of Iceberg lettuce stored at different temperatures (2 °C, 5 °C, 10 °C, 15 °C and 20 °C) during shelf life are presented in Table 3. No remarkable differences in TA were found between the initial and last day of storage at each temperature. 

The SSC and pH were not affected by high temperatures (20, 15 and 10 °C) during short storage periods. A decrease of 24% and 23.1% in SSC at 5 and 2 °C respectively in Iceberg lettuce was observed during long storage periods (21 and 29 days respectively). Although the TA remained constant, the pH increased by 4.0% at 5 °C in Iceberg lettuce during long storage periods (21 and 29 days respectively). 

**Figure 4:**
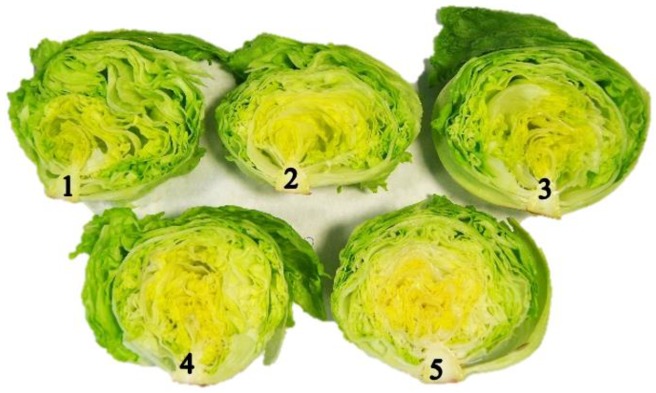
Different compactness stages in Iceberg Lettuce: 1: Soft, easily compressible; 2: Slightly firm. Good head formation; 3: Firm. Compact but can be broken with light or moderate pressure; 4: Hard. Compact and solid; 5: Extra-hard. Over-mature, there may be broken central ribs [44].

Due to the structure, the firmness of Iceberg lettuce heads was 4.84 and 2.64 N mm^−1^. These values are similar to those reported by Martínez-Romero [42]. The firmness was not affected by temperature or storage time conditions. However, greater standard deviations were observed in lettuce since the state of compactness was different for each lettuce. The different stages observed in this trial are presented in Figure 4 which is in concordance with what other authors have also reported [35]. The ΔE of the lettuce heads was not affected in any of the storage temperatures tested.

**Figure 5:**
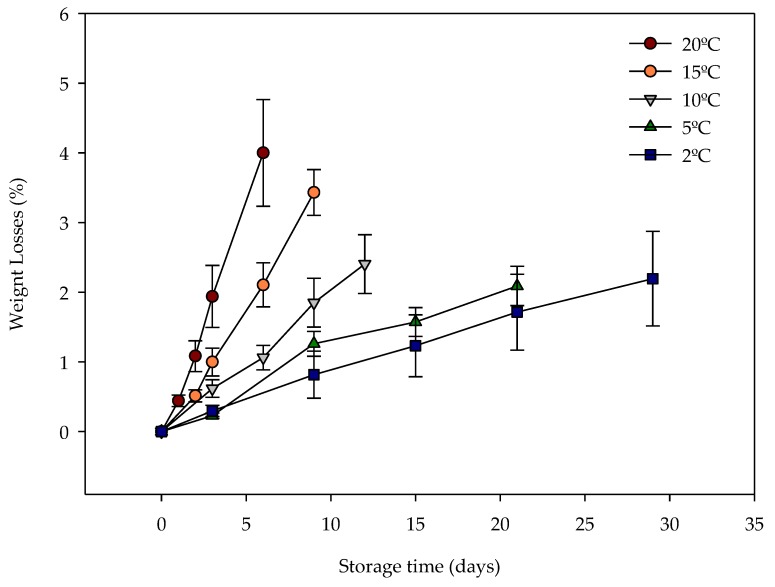
Evolution of weight losses for iceberg lettuce during shelf life at several storage temperatures.

The weight loss was greater on the last sampling day for each temperature, reaching 4.0/1.64%, 3.4/1.4%, 2.4/1.26%, 2.1/0.65% and 2.2/2.9% at 20 °C, 15 °C, 10 °C, 5 °C and 2 °C respectively (Figure 5).

Weight loss is associated with quality loss in lettuce. For this reason, Table 4 presents the correlation coefficients of weight loss with some subjective quality parameters that can be evaluated when lettuce is received in a packinghouse. Both parameters, visual appearance and overall quality obtained significant high correlation coefficients (*p* ≤ 0.001). The correlations were negative, which indicates that a greater loss of weight is detrimental to the quality parameters.

The commodity score is calculated by relating several multi-quality indices [45,46]. In this work, subjective quality parameters have been categorized in the sensory analysis using the methodology proposed in Section 2.2.4. Table 5 presents the final data for overall acceptance.

**Figure 6:**
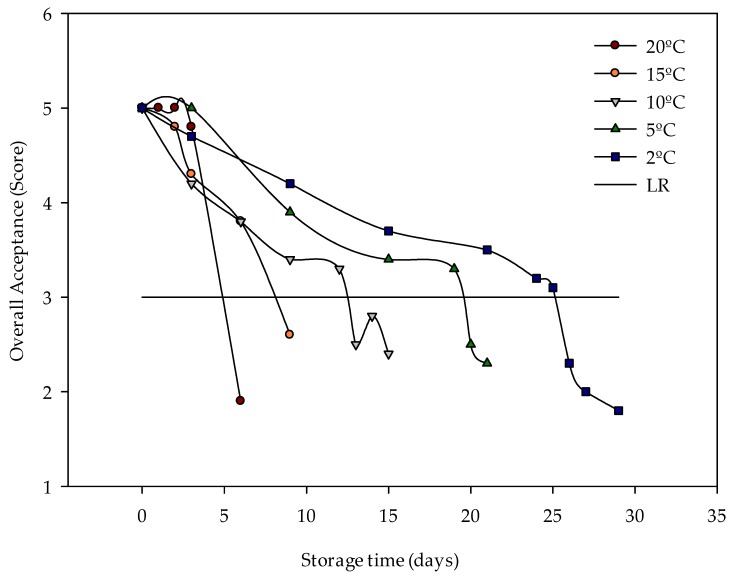
Changes in global acceptability of iceberg lettuces stored at a constant temperature. Scale: 5: excellent; 4: good; 3: fair – limit of retail (LR); 2: poor; 1: extremely bad.

Figure 6 shows the evolution of the global acceptability score in the different analyses performed at a constant temperature. Table 6 presents the shelf life obtained after temperature-controlled trials in cold storage rooms. The cut-off date was determined as the last day on which overall acceptance was greater than 3 (Score 3).

### 3.2. Land Transportation Trials

Land transportation trials were carried out to determine how the air temperature varies during 3–5 day shipments. Sensors were installed before the loading process. Other trials were performed in cold storage rooms simulating land transport conditions. In these trials, the temperature was changed during the first 4 or 5 days with variations similar to those registered in the land transportation experimental trials. Both in cold storage rooms and land transportation, the temperature was measured by the sensor nodes. The variation of the set-point was determined due to refrigeration [47] or the frost-free system of the container, or even due to the distribution of the goods within the container. 

**Figure 7:**
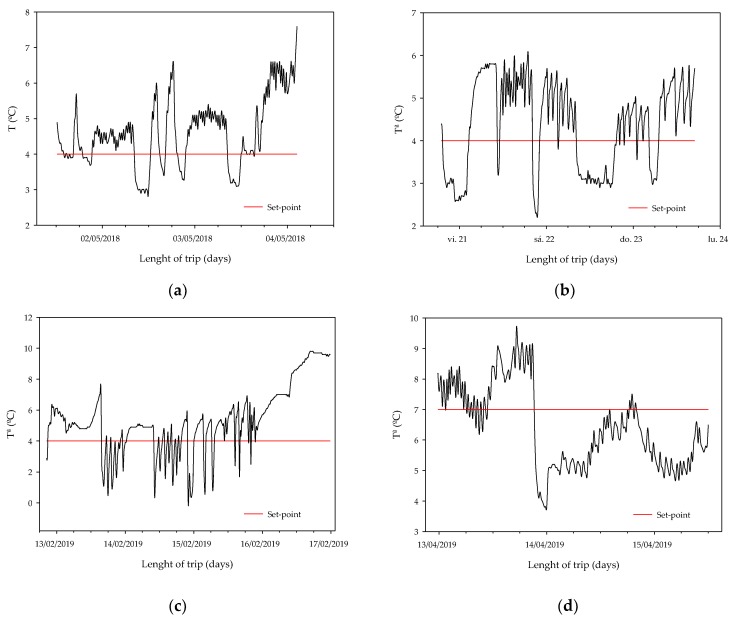
Time-temperature data recorded in real-time by the gateway sensor node in diverse journeys (**a**) Germany, (**b**) The Netherlands, (**c**) The United Kingdom and (**d**) Belgium. The horizontal line is the set-point temperature.

As presented in Figure 3, gateway sensor nodes were placed on the roof of the container. Figure 7 illustrates the temperature registered by the gateway sensor node for the four previous travels summarized, indicating the set-point temperature with the horizontal line. The frost-free system of the container causes the temperature variation, describing opposed peaks that can be observed in the graphs. In these cases, the distribution of the nodes should not be a condition because they were always placed in the same position and the load distribution was the same in all the trips, (33 euro-pallets).

Differences between trips are observed. Figure 7a shows a three-day trip with a set-point temperature of 4 °C, as Figure 7b, but with a bigger temperature fluctuation for a greater range of temperatures between 2.9 °C and 7.6 °C, while in Figure 7b even with the frost-free system there are temperature differences of up to 3.8 °C. 

Figure 7c shows less temperature difference until the container starts to get warm, but it is under the set-point temperature for a greater length of time than for the other trip. The variation is shown in Figure 7d is the most significant compared to the others, where the peak on the graph may correspond to adjusted storage temperature, or just to the frost-free system causing a temperature drop of 3.2 °C from the set-point temperature of 7 °C.

There is one thing they all have in common, while the frost-free system is not working, they show small fluctuations which not should be relevant to the quality of the commodities for a short trip. 

### 3.3. Features and Mathematical Models

Features were extracted from the sensors both in real land transportations and cold storage rooms simulations, named “dummy” in Table 7.

Three multivariable regression models have been tested:

Model 1, MNLR with Interactions

Relevant observations: set-point (SP) and the area above set-point (A)
Term*β*SET-value*p*-valueVIFConstant29.0050.38974.560.000
SP−2.18710.0981−22.300.00019.00A0.16540.04613.590.003353.58SP^2^0.049940.0045311.010.00018.65SP*A−0.037210.00920−4.040.001353.34

*Days with score 3* = 29.005 − 2.1871 × *SP* + 0.1654 × *A* + 0.04994 × *SP*^2^ − 0.03721 × *SP × A* (5)

SR^2^R^2^ adjustedR^2^ predicted0.43406099.51%99.36%98.37%

Model 2, MLR with Interactions

Relevant observations: set-point (SP) and area above set-point (A)
Term*β*SET-value*p*-valueVIFConstant25.7950.66738.700.000
SP−1.17850.0787−14.970.0001.04A−0.024920.00857−2.910.0101.04

*Days with score 3* = 25.795 − 1.1785 × *SP* − 0.02492 × *A* (6)

SR^2^R^2^ adjustedR^2^ predicted1.4905193.34%92.51%85.98%

Model 3, MNLR without Interactions

Relevant observations: set-point (SP) and area above set-point (A)
Term*β*SET-value*p*-valueVIFConstant29.6040.51257.850.000
SP−2.3130.132−17.480.00017.09A−0.020630.00358−5.760.0001.06SP^2^0.054920.006218.840.00017.28

*Days with score 3* = 29.604 − 2.313 × *SP* − 0.02063 × *A* + 0.05492 × *SP*^2^ (7)

SR^2^R^2^ adjustedR^2^ predicted0.61753698.93%98.71%97.45%

**Figure 8:**
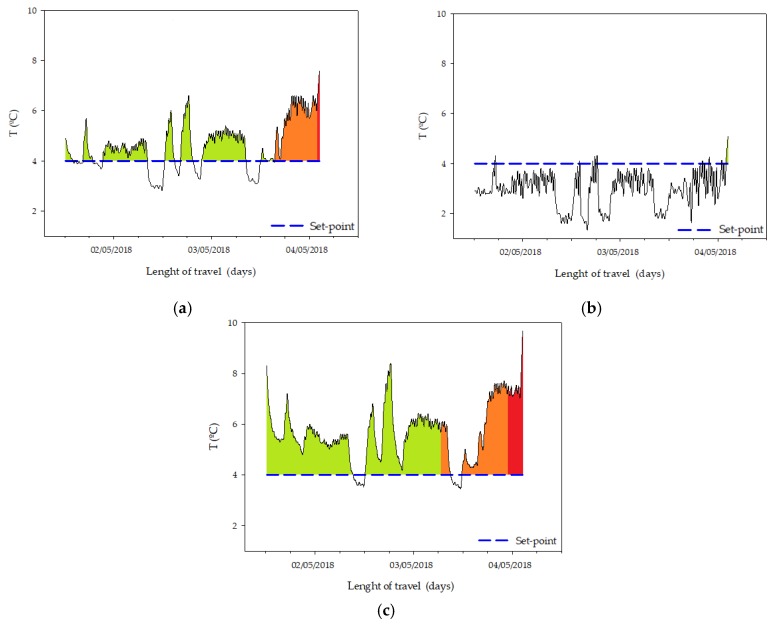
Register of the historical temperature recorded in several locations inside of the same truck (**a**) Gateway, (**b**) Slave 1 and (**c**) Slave 2. The nodes were placed as is shown in Figure 3.

These results can be applied as an example of the temperature distribution inside of a trailer at land transportation. The losses are shown in Table 8 and their areas are evaluated in Figure 8.

Concerning the temperature distribution inside the transport, Figure 8 presents the variations of the temperature among the different locations of the sensors (gateway and slave nodes), according to Figure 3. 

The results of the proposed model are represented by different colours. A green colour indicates that the registered variation of temperature is not enough to reduce the quality of the load, orange colour means that the shelf life is probably getting reduced and red colour means that the registered conditions have reduced the theoretical shelf life.

## 4. Discussion and Conclusions

Cantwell and Suslow [35] reported that lettuces have moderate respiration rates and they are more vulnerable to temperature changes and weight loss, as their metabolism accelerates with increasing temperature. The firmness of the lettuce heads was constant throughout their shelf life without differences between the storage temperatures tested. The studied lettuce heads had different compactness stages, and this could influence firmness values. Deza-Durand and Petersen [48] highlighted the complex structure of lettuce composed of photosynthetic and vascular tissues whose metabolism differs between the internal and external leaves of the same lettuce head.

Weight loss of iceberg lettuces increased each sampling day. Lettuce stored at higher temperatures resulted in greater weight loss. However, moderate weight loss was observed for all storage temperatures compared to Managa et al. [49], who reported 45.31% weight loss in Iceberg lettuce stored for 3 days at room temperature. According to Manolopoulou et al. [50], this may be due to the high relative humidity generated around the head of lettuce when they are bagged. No visual dehydration was observed in the ‘human’ sensory analyses.

The quality parameters were negatively influenced by temperature increases. The overall quality of the lettuce heads was above the shelf life limit except on the last day of storage for each storage temperature. However, on the last day of storage, the lettuce heads appeared to be in good condition to be consumed, but not to be marketed and sold to a consumer who will still need at least 4–5 days to eat them. Slight shrivelling was observed on the external leaves due to the greater exposure of such leaves to environmental factors that facilitate their deterioration. As a consequence, the internal zone is protected and its visual appearance is better preserved [51].

During the trips, the temperature is not the same in any node. Furthermore, it is reported that temperature is not always regularly spread inside the pallet, and one single sensor for the entire pallet does not provide a realistic distribution [52]. Jedermann et al. [47] reported that the thermal mass of the commodities influences the differences between the ambient temperature and the temperature of the pallet core. In addition, the differences between the nodes during the same trip are influenced by their position in the container [51,52]. This was clearly shown during the first trip registered to Germany (Table 8 and Figure 8a–c). The purpose of this work is not to make an exhaustive study of the shelf life, but to provide an estimation model using the technical means currently used in the land transportation of lettuce, such as air temperature measurement. The presented system allows, to the different intermediaries, to know the conditions in which the load have been stored and/or transported and could contribute to reducing the food waste due to bad conditions of conservation or transportation. However, in order to make a more accurate quality loss prediction model, it is necessary to consistently extend the training and validation work throughout the time, not only after transportation but also in the different phases of the cold chain. This target should be evaluated in future works.

Concerning the regression models, the best results were obtained using the temperature of the set-point and the area above. The average temperature is not decisive in the prediction. The temperature variation observed in the tests corresponds to certain phases of loading or unloading the lettuce into the truck or, in the case of the simulated tests, the transportation of the goods from the handling industry to the cold storage rooms at university. In all situations, the PCA analysis has shown that these temperature variations are not relevant due to their short duration. Besides, both the area and the set-point are variables that are measured in real-time, so the estimation of the shelf life can be done in advance by observing these values.

Model 1 is the model that best correlates with the results. It predicts very well the duration in days and the errors are always in a conservative sense (it proposes a duration lower than that observed) with a maximum error of 1 day. However, it has a complicated interpretation.

Model 2 is easily interpreted. Each increased degree causes a loss of 1.17 days of duration and a unit increase of the area also produces a decrease of 0.025 days. Makes mistakes in both directions, in a range of 3 to 2 days. The model may have some problem since there is a decrease in the R^2^ predictor relating to the others.

In model 3, the quadratic term of the area is not significant. This model conservatively makes mistakes, in a range of 0 to 2 days.

Although the model 1 and 3 have similar behaviour, model 3 is much easier to interpret than model 1, since an increase in the value of predictor A (Area) leads to a reduction in shelf life. Thus, in model 3, the coefficient corresponding to predictor A is negative (−0.02063), while in model 1 it is positive (0.1654), in the latter case compensating the duration with the negative coefficient of the interaction (−0.03721). In summary, applying the criterion of statistical parsimony, model 3 is the most appropriate since it presents an explanatory level very similar to that obtained with model 1 using a smaller number of predictors.

In conclusion, the area above the set-point can be considered a relevant observation that allows predicting quality loss during storage or in-transit refrigerated food. These observations allow using the system presented as a ‘Shelf life estimator sensor’. The more data tested, the better the model can predict quality and remaining shelf life. In this way, the system can be used in multiple scenarios of the supply chain, which means that it is a very versatile tool able to adapt to the behaviour of the commodities under different environmental conditions along the supply chain for estimated shelf life. We concluded that these sensor-based tools can be very useful in helping to reduce food losses. For instance, product rejection after poor temperature preservation during transportation can be avoided knowing the estimated remaining shelf life. These tools can help to decide to send such commodities to end-users with shorter shelf life or to supermarkets with high commodity turnover.

In future researches, this tool should be implemented. It should be able to predict the remaining shelf life for all kinds of commodities according to the initial quality and the environmental factors affecting the location where they have been stored or transported from farm to fork. It should also include non-sensory quality attributes such as, i.e. nutritional compounds, like vitamin C content loss, which is being increasingly demanded by consumers for fresh food.

## 5. Patents

The system is patented by the authors in Spain (‘Dispositivo, sistema y método de monitorización en tiempo real de las variables físicas y ambientales durante el transporte de mercancías perecederas’; Registration number: ES201730301A). 

## Figures and Tables

**Table 1:** Shelf life, quality sampling days and number of lettuces tested per set according to the storage temperature.

Storage Temperature (°C)	Recommended Shelf Life (days)	Quality Analysis Days	Number of Tested Lettuces Per Set
20	6	0, 1, 2, 3, 6	25
15	9	0, 2, 3, 6, 8, 9, 10	35
10	15	0, 3, 6, 9, 14, 15, 16	35
5	21	0, 3, 9, 15, 20, 21, 22	35
2	29	0, 3, 9, 15, 21, 28, 29, 30	40

**Table 2:** Respiration rates (mL CO_2_/kg h) of Iceberg lettuce.

Temperature	2 °C	5 °C	10 °C
Iceberg	5.2 ± 1.1	8.0 ± 1.7	17.5 ± 3.3

**Table 3:** Physico-chemical parameter results of iceberg lettuce analysis ^1^.

T^a^	Day	SSC (°Brix)	pH	TA (g Citric Acid L−1)	Firmness (N mm−1)	ΔE
20 °C	0	$4.16\pm 0.17_{\mathrm{ab}}^{A}$	$6.04\pm 0.06_{a}^{A}$	$0.68\pm 0.09_{\mathrm{ab}}^{A}$	$4.84\pm 3.29_{a}^{A}$	-
	1	$3.58\pm 0.06_{c}$	$6.02\pm 0.06_{a}$	$0.61\pm 0.06_{b}$	$4.36\pm 1.19_{a}$	$2.69\pm 2.56_{a}$
	2	$4.60\pm 0.61_{a}^{A}$	$6.02\pm 0.00_{a}^{B}$	$0.78\pm 0.06_{a}^{A}$	$3.78\pm 0.69_{a}^{A}$	$4.55\pm 2.30_{a}^{A}$
	3	$3.96\pm 0.17_{\mathrm{bc}}^{A}$	$5.98\pm 0.06_{a}^{C}$	$0.68\pm 0.12_{\mathrm{ab}}^{\mathrm{AB}}$	$3.74\pm 0.73_{a}^{B}$	$2.28\pm 0.70_{a}^{A}$
	6	$4.16\pm 0.06_{\mathrm{ab}}^{A}$	$6.04\pm 0.06_{a}^{A}$	$0.73\pm 0.09_{\mathrm{ab}}^{A}$	$4.79\pm 1.98_{a}^{A}$	$2.83\pm 1.81_{a}^{A}$
15 °C	0	$4.16\pm 0.17_{\mathrm{ab}}^{A}$	$6.04\pm 0.06_{a}^{A}$	$0.68\pm 0.09_{a}^{A}$	$4.84\pm 3.29_{a}^{A}$	-
	2	$3.80\pm 0.25_{\mathrm{ab}}^{B}$	$6.10\pm 0.00_{a}^{A}$	$0.64\pm 0.04_{a}^{A}$	$3.97\pm 1.31_{a}^{A}$	$2.22\pm 1.08_{\mathrm{ab}}^{A}$
	3	$4.34\pm 0.47_{a}^{A}$	$6.04\pm 0.06_{a}^{\mathrm{BC}}$	$0.71\pm 0.04_{a}^{A}$	$5.35\pm 2.01_{a}^{\mathrm{AB}}$	$3.17\pm 1.98_{\mathrm{ab}}^{A}$
	6	$4.22\pm 0.42_{\mathrm{ab}}^{A}$	$6.08\pm 0.06_{a}^{A}$	$0.63\pm 0.03_{a}^{A}$	$4.88\pm 1.36_{a}^{A}$	$4.43\pm 1.78_{a}^{A}$
	9	$3.54\pm 0.35_{b}^{A}$	$6.10\pm 0.00_{a}^{B}$	$0.66\pm 0.04_{a}^{A}$	$4.26\pm 0.65_{a}^{A}$	$1.57\pm 1.20_{b}^{A}$
10 °C	0	$4.16\pm 0.17_{a}^{A}$	$6.04\pm 0.06_{a}^{A}$	$0.68\pm 0.09_{a}^{A}$	$4.84\pm 3.29_{a}^{A}$	-
	3	$3.76\pm 0.47_{a}^{A}$	$6.04\pm 0.06_{a}^{\mathrm{AB}}$	$0.58\pm 0.02_{a}^{\mathrm{BC}}$	$5.86\pm 2.33_{a}^{\mathrm{AB}}$	$2.16\pm 1.31_{a}^{A}$
	6	$3.52\pm 0.26_{a}^{B}$	$6.10\pm 0.00_{a}^{A}$	$0.64\pm 0.03_{a}^{A}$	$4.07\pm 0.70_{a}^{A}$	$4.09\pm 2.80_{a}^{A}$
	9	$3.58\pm 0.40_{a}^{A}$	$6.10\pm 0.00_{a}^{B}$	$0.63\pm 0.07_{a}^{A}$	$4.73\pm 1.26_{a}^{A}$	$3.34\pm 1.27_{a}^{A}$
	15	$3.62\pm 0.10_{a}^{A}$	$6.12\pm 0.06_{a}^{A}$	$0.62\pm 0.11_{a}^{B}$	$4.32\pm 0.76_{a}^{A}$	$5.37\pm 1.38_{a}^{A}$
5 °C	0	$4.16\pm 0.17_{a}^{A}$	$6.04\pm 0.06_{c}^{A}$	$0.68\pm 0.09_{a}^{A}$	$4.84\pm 3.29_{a}^{A}$	-
	3	$4.18\pm 0.00_{a}^{A}$	$6.20\pm 0.00_{\mathrm{ab}}^{A}$	$0.58\pm 0.03_{a}^{\mathrm{BC}}$	$3.94\pm 2.26_{a}^{B}$	$3.33\pm 2.08_{a}^{A}$
	9	$3.88\pm 0.15_{a}^{A}$	$6.16\pm 0.00_{b}^{B}$	$0.61\pm 0.03_{a}^{A}$	$4.13\pm 1.78_{a}^{A}$	$2.97\pm 1.56_{a}^{A}$
	15	$3.88\pm 0.26_{a}^{A}$	$6.20\pm 0.00_{\mathrm{ab}}^{A}$	$0.46\pm 0.07_{b}^{A}$	$3.87\pm 0.67_{a}^{\mathrm{AB}}$	$3.09\pm 2.19_{a}^{\mathrm{AB}}$
	21	$3.16\pm 0.31_{b}^{A}$	$6.28\pm 0.00_{a}^{A}$	$0.61\pm 0.03_{a}^{A}$	$6.16\pm 3.41_{a}^{A}$	$2.75\pm 1.48_{a}^{A}$
2 °C	0	$4.16\pm 0.17_{a}^{A}$	$6.04\pm 0.06_{c}^{A}$	$0.68\pm 0.09_{a}^{A}$	$4.84\pm 3.29_{\mathrm{ab}}^{A}$	-
	3	$3.80\pm 0.20_{\mathrm{ab}}^{A}$	$6.26\pm 0.00_{a}^{A}$	$0.55\pm 0.02_{\mathrm{bc}}^{C}$	$7.59\pm 0.00_{a}^{A}$	$3.80\pm 2.47_{a}^{A}$
	9	$3.66\pm 0.00_{\mathrm{bcd}}^{A}$	$6.24\pm 0.00_{\mathrm{ab}}^{A}$	$0.53\pm 0.03_{c}^{B}$	$2.83\pm 0.80_{b}^{A}$	$2.20\pm 0.70_{a}^{A}$
	15	$3.70\pm 0.35_{\mathrm{abc}}^{A}$	$6.12\pm 0.06_{\mathrm{bc}}^{A}$	$0.64\pm 0.04_{a}^{A}$	$2.84\pm 0.72_{b}^{B}$	$1.80\pm 0.68_{a}^{B}$
	21	$3.32\pm 0.06_{\mathrm{cd}}^{A}$	$6.28\pm 0.06_{a}^{A}$	$0.63\pm 0.02_{\mathrm{ab}}^{A}$	$4.21\pm 1.06_{b}^{A}$	$2.55\pm 1.70_{a}^{A}$
	29	$3.20\pm 0.40_{d}$	$6.16\pm 0.06_{\mathrm{abc}}$	$0.66\pm 0.04_{a}$	$3.68\pm 1.58_{b}$	$4.04\pm 2.39_{a}$

^1^ Different capital letters denote significant differences (*p* ≤ 0.05) among temperatures for the same sampling day. Different lowercase letters denote significant differences (*p* ≤ 0.05) among sampling days for the same temperature.

**Table 4:** Coefficients of linear correlation for weight loss with sensory quality parameters (visual appearance, odour, colour and overall quality) during different storage temperatures in Iceberg lettuce.

	Subjective Quality Parameters			
	Visual Appearance	Odour	Colour	Overall Quality
20 °C	−0.733	−0.812	−0.859	−0.851
15 °C	−0.953	−0.564	−0.788	−0.910
10 °C	−0.795	−0.740	−0.765	−0.786
5 °C	−0.819	−0.811	−0.804	−0.924
2 °C	−0.756	−0.756	−0.794	−0.7119

**Table 5:** Overall acceptance (score) after all parameters have been rated.

20 °C	STORAGE DAY	0	1	2			3	5		6
	Overall acceptance	5.0	5.0	5.0			4.8	3.6		1.9
15 °C	Storage day	0	2	3	6		7	8		9
	Overall acceptance	5.0	4.8	4.3	3.8		3.4	3.1		2.6
10 °C	Storage day	0	3	6	9	11	12	13	14	15
	Overall acceptance	5.0	4.2	3.8	3.4	3.3	3.2	2.5	2.8	2.4
5 °C	Storage day	0	3	9	15	17	18	19	20	21
	Overall acceptance	5.0	5.0	3.9	3.4	3.4	3.3	3.3	2.5	2.7
2 °C	Storage day	0	3	9	15	21	24	25	26	27
	Overall acceptance	5.0	4.7	4.2	3.7	3.5	3.2	3.1	2.3	2.0

**Table 6:** The shelf life of Iceberg lettuces according to the fixed storage temperatures.

Temp. (°C)	Shelf Life (Days)
20	5
15	8
10	12
5	19
2	25

**Table 7:** Temperature information registered both land transportations and the cold storage rooms.

Destination	Days of Travel	Setpoint Temperature (°C)	ΔT^a^ (max/min) (°C)	Average T^a^ (°C)	Area Above Set-Point
Germany	3	4	8.2	4.4	22.7
The Netherlands	3	4	6.4	4.5	28.7
United Kingdom	4	5	9.7	5.5	11.3
Belgium	3	7	7	7.1	14.9
Germany	3	4	5	4.1	18.6
Dummy 1	4	5	7.6	7.2	171.3
Dummy 2	4	5	12.5	5.1	36.8
Dummy 3	5	5	10.6	5.1	55.7
Dummy 4	5	10	1.7	10.5	9.5
Dummy 5	5	5	1.8	5	3.6
Dummy 6	5	2.5	2.4	2.5	4.2

**Table 8:** Shelf life differences among the diverse locations inside the truck. The nodes were placed as is shown in Figure 3. Model 3 was implemented to predict the days lost.

Destination	Set T^a^ (°C)	Days Lost Gateway	Days Lost Slave 1	Days Lost Slave 2
Germany	4	0	0	1
The Netherlands	4	0	-	0
United Kingdom	5	1	-	1
Belgium	7	0	0	0
